# Structure of the WIPI3/ATG16L1 Complex Reveals the Molecular Basis for the Recruitment of the ATG12~ATG5-ATG16L1 Complex by WIPI3

**DOI:** 10.3390/cells13242113

**Published:** 2024-12-20

**Authors:** Xinyu Gong, Yingli Wang, Yuqian Zhou, Lifeng Pan

**Affiliations:** 1 State Key Laboratory of Chemical Biology, Shanghai Institute of Organic Chemistry, University of Chinese Academy of Sciences, Chinese Academy of Sciences, Shanghai 200032, China; gongxinyu@sioc.ac.cn (X.G.); wangyingli@sioc.ac.cn (Y.W.); 13602101879@163.com (Y.Z.); 2 School of Chemistry and Materials Science, Hangzhou Institute for Advanced Study, University of Chinese Academy of Sciences, 1 Sub-Lane Xiangshan, Hangzhou 310024, China

**Keywords:** macroautophagy, autophagy, ATG16L1, WIPI3, alternative autophagy

## Abstract

Macroautophagy deploys a wealth of autophagy-related proteins to synthesize the double-membrane autophagosome, in order to engulf cytosolic components for lysosome-dependent degradation. The recruitment of the ATG12~ATG5-ATG16L1 complex by WIPI family proteins is a crucial step in autophagosome formation. Nevertheless, the molecular mechanism by which WIPI3 facilitates the recruitment of the ATG12~ATG5-ATG16L1 complex remains largely unknown. Here, we uncover that WIPI3 can directly interact with the coiled-coil domain of ATG16L1. By determining the crystal structure of WIPI3 in complex with ATG16L1 coiled-coil, we elucidate the molecular basis underpinning the specific recruitment of the ATG12~ATG5-ATG16L1 complex by WIPI3. Moreover, we demonstrate that WIPI2 and WIPI3 are competitive for interacting with ATG16L1 coiled-coil, and ATG16L1 and ATG2 are mutually exclusive in binding to WIPI3. In all, our findings provide mechanistic insights into the WIPI3/ATG16L1 interaction, and are valuable for further understanding the activation mechanism of the ATG12~ATG5-ATG16L1 complex as well as the working mode of WIPI3 in autophagy.

## 1. Introduction

Macroautophagy (hereafter autophagy) entails the de novo synthesis of the double-membrane autophagosome, which originates from the phagophore and eventually fuses with the lysosome, for fulfilling the degradation and recycling of autophagic cargoes, such as pathogenic protein aggregates, damaged organelles, and infectious pathogens [1,2,3,4,5,6]. In this manner, autophagy maintains cellular and organismal homeostasis, and is crucial for numerous physiological processes, such as cell differentiation, cell death, immune response, and aging [6,7]. Importantly, dysfunctions of autophagy are associated with multitudinous human diseases, including cancer, neurodegenerative diseases, and Crohn’s disease [3,4,8].

In canonical autophagy, the nucleation and elongation processes of the phagophore rely on the coordinated actions of a series of autophagy-related (ATG) proteins [4]. In particular, the FAK family kinase-interacting protein of 200 kDa (FIP200) responds to the calcium transients, and undergoes the liquid–liquid phase separation as well as the Unc-51-like kinase (ULK) complex, which is followed by the translocation and phosphorylation of the class III phosphatidylinositol 3-kinase complex I (PI3KC3-C1) [9]. Then, the generation of phosphatidylinositol-3-phosphate (PI3P) from activated PI3KC3-C1 can be specifically perceived and accelerated by the WD repeat domain phosphoinositide-interacting (WIPI) family proteins, including WIPI1, WIPI2, WIPI3, and WIPI4 in mammals [10,11]. Subsequently, WIPI2 employs a distinctive dual-binding site mechanism to recruit and activate the ATG12~ATG5-ATG16L1 complex (hereafter referred to as ATG16L1 complex) that catalyzes the phosphatidylethanolamine (PE) lipidation of ATG8 family protein with the limited assistance of WIPI1 [12,13,14,15]. Meanwhile, the PE-decorated ATG8 family proteins further recruit the WIPI3/4-ATG2 complex to supply lipid for phagophore expansion [16].

**Figure 1:**
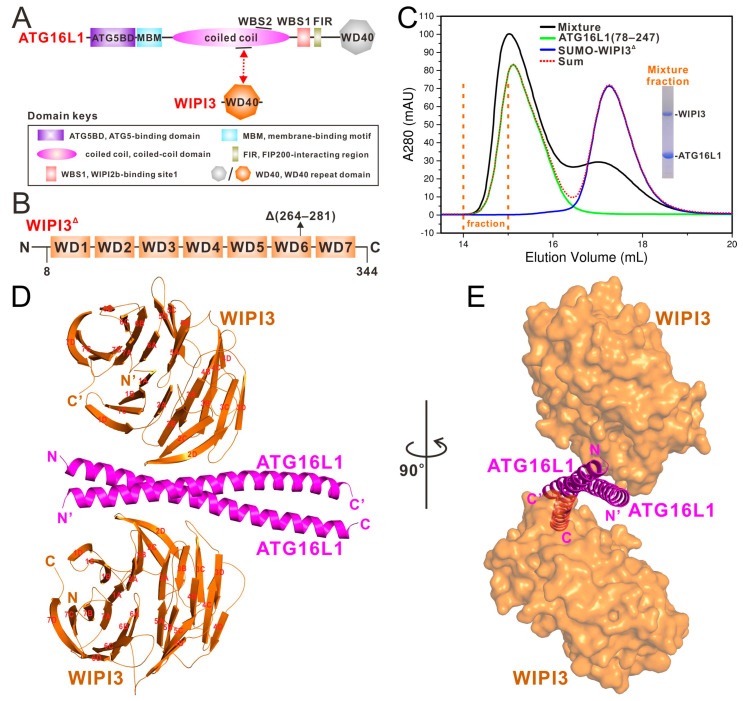
Biochemical and structural characterizations of the interaction between ATG16L1 and WIPI3. (**A**) The schematic diagram showing the domain organizations of ATG16L1 and WIPI3. In this drawing, the interaction of ATG16L1 with WIPI3 is highlighted and indicated by a two-way arrow. (**B**) The schematic diagram of WIPI3^Δ^ including residues 8–344 but without residues 264–281. (**C**) Size exclusion chromatography-based analyses of the interaction of ATG16L1(78–247) with SUMO-tagged WIPI3^Δ^. In this panel, “Sum” stands for the theoretical sum of ATG16L1(78–247) and SUMO-WIPI3^Δ^ profiles, while “Mixture” stands for the ATG16L1(78–247) and SUMO-WIPI3^Δ^ mixture sample. The insert in the panel shows the SDS-PAGE combined with Coomassie-blue staining analysis of the protein components of the indicated “Mixture fraction” fraction collected from the size exclusion chromatography experiment of the “Mixture” sample (the black curve). (**D**,**E**) The combined ribbon and surface representation diagram showing the overall structure of the WIPI3^Δ^/ATG16L1 complex. In this drawing, WIPI3^Δ^ and ATG16L1 are shown in orange and magenta, respectively.

As a key autophagic protein, ATG16L1 interacts with ATG12~ATG5 conjugate through its N-terminal ATG5-binding domain (ATG5BD) (Figure 1A), and further assembles into the hetero-hexameric E3-like ATG16L1 complex, utilizing its dimeric coiled-coil domain [17,18]. Notably, ATG16L1 typically determines the localization of the ATG16L1 complex, thereby specifying the lipidation site of ATG8 family proteins [19]. In canonical autophagy, the recruitment of ATG16L1 depends on the specific associations between the central region of ATG16L1 and multiple autophagic upstream factors, such as WIPI2 [13,14]. Meanwhile, the seven-bladed WD40 domain of human ATG16L1 (Figure 1A), which is absent in the yeast homolog ATG16, plays crucial roles in the conjugation of ATG8 to single membranes (CASM) [20]. Interestingly, in contrast to that of WIPI2 or WIPI3, yeast ATG21 enables the recruitment of human ATG16L1 complex but does not promote the lipidation of ATG8 family proteins despite the unknown mechanism [11], suggesting that the recruitment of the ATG16L1 complex alone is insufficient for mediating the subsequent lipidation process. Concurrently, WIPI3 can replace WIPI2 to fulfill the positive feedback with PI3KC3-C1 and promote the lipidation of ATG8 family proteins [11]. Structurally, both WIPI3 and WIPI2 are mainly composed of a seven-bladed WD40 domain that contains a highly conserved “L/FRRG” motif to sense PI3P and a unique loop connecting the two outermost β-strands of blade 6 for membrane binding [14,21,22]. However, in comparison to that of WIPI2, how WIPI3 specifically recognizes and promotes the activity of the ATG16L1 complex remains largely unknown.

In addition to canonical autophagy, accumulating evidences uncovered the existence of alternative autophagy [23,24], which degrades undelivered secretory cargoes such as insulin granules [25], eliminates mitochondria from erythrocytes [23], protects intestinal epithelial cells from bacterial infection [26], and prevents TNF cytotoxicity [27]. Alternative autophagy shares the same autophagy initiation machinery, including the ULK complex and PI3KC3-C1, with canonical autophagy. But, intriguingly, alternative autophagy normally functions in the absence of WIPI2 or the ATG16L1 complex, and depends on WIPI3 that senses the PI3P signal from PI3KC3-C1 and brings along ATG2, which transports essential lipids for autophagosome formation [23,28]. Nevertheless, why WIPI3 does not recruit the ATG16L1 complex during alternative autophagy is still elusive, and the detailed relationship between the ATG16L1 complex and ATG2 in binding to WIPI3 remains to be elucidated.

In this study, we systemically characterize the binding between WIPI3 and the ATG16L1 complex, and discover that WIPI3 directly interacts with the coiled-coil region of ATG16L1. The determined crystal structure of WIPI3 in complex with the ATG16L1 coiled-coil domain not only uncovers the detailed molecular basis underlying the specific association of WIPI3 with ATG16L1, but also provides a mechanistic insight into the activation of the ATG16L1 complex. Finally, we demonstrate that WIPI3 and WIPI2 are competitive for associating with ATG16L1, and ATG16L1 and ATG2 are mutually exclusive in binding to WIPI3, thereby providing novel insights into the molecular basis of WIPI3-mediated alternative autophagy.

## 2. Materials and Methods

### 2.1. Materials

The HEK293T and HeLa cell lines are from Prof. Junying Yuan from the Interdisciplinary Research Center on Biology and Chemistry, CAS, Shanghai, China. The full-length human *ATG5*, *ATG12*, *ATG16L1,* and *WIPI2* genes were obtained from Prof. Jiahuai Han from the School of Life Sciences, Xiamen University, China. The full-length human *WIPI3* gene was amplified from a cDNA library that was constructed from HeLa cells through the PCR method.

For protein expression using *E. coli* cells, human WIPI3 (residues 8–344 without 264–281) and WIPI2b (residues 13–362 without 265–297) were fused with an N-terminal SUMO or GST tag. Human ATG16L1 (residues 78–247, 78–206, 78–197, 124–197, and 124–188) and ATG2A (residues 1378–1402) were all fused with an N-terminal Trx tag. Full-length human ATG5 and ATG12 were fused with an N-terminal Trx tag and MBP tag, respectively. For co-immunoprecipitation assays, full-length human WIPI3 and ATG16L1 were separately fused with an N-terminal Flag tag and mEGFP tag (mEGFP contains an A206K mutation at EGFP), respectively. All point mutations of WIPI3 and ATG16L1 used in this study were generated through the standard PCR-based mutagenesis method.

### 2.2. Protein Expression and Purification

Recombinant proteins were all expressed for 15~18 h in BL21 (DE3) *E. coli* cells induced by 200 μM IPTG at 16 °C. The bacterial cells were collected by centrifugation and re-suspended in the buffer containing 50 mM Tris (pH = 7.9), 500 mM NaCl, and 5 mM imidazole, and then lysed using the ultrahigh pressure method. Then, the lysate was spun down by centrifuge at 17,000 rpm (35,000× *g*) for 35 min to collect the supernatant. Then, proteins were purified using the lysate supernatant through Ni^2+^-NTA agarose (GE Healthcare, USA) affinity chromatography followed by the use of a Superdex 200 column (GE Healthcare, USA) equilibrated with the freshly prepared column buffer containing 20 mM Tris (pH = 7.5), 100 mM NaCl, and 1 mM DTT.

### 2.3. Isothermal Titration Calorimetry (ITC) Assay

ITC measurements were all carried out on a MicroCal PEAQ-ITC (Malvern, UK) calorimeter at 25 °C. All protein samples were freshly purified in the same column buffer containing 20 mM Tris (pH = 7.5), 100 mM NaCl, and 1 mM DTT. The relevant concentrated 50 μM and 500 μM protein samples were prepared for the cell and the syringe of the ITC calorimeter, respectively. In each ITC experiment, protein samples from the syringe were sequentially injected into the cell at time intervals of 2 min.

### 2.4. Size Exclusion Chromatography

The purified proteins were firstly subjected to centrifugation at 14,500× *g* for 15 min at 4 °C, after which they were loaded onto a Superdex 200 Increase 10/300 GL or Superdex 75 10/300 GL column (GE Healthcare, USA). All the experiments were conducted on an AKTA FPLC system (GE Healthcare, USA), and the relevant results were exported and further analyzed using Origin 9 software.

### 2.5. Protein Crystallization and Structural Elucidation

The crystals of the WIPI3^Δ^/ATG16L1(124–188) complex were obtained using the sitting-drop vapor-diffusion method at 16 °C, under the crystal-growing condition containing 0.02 M Citric acid, 0.08 M BIS-TRIS propane (pH = 8.8), and 16% *w*/*v* Polyethylene glycol 3350. A 2.77 Å resolution X-ray diffraction data set for the WIPI3^Δ^/ATG16L1(124–188) complex was collected at the beamline BL10U2 or BL19U1 of the SSRF [29]. The diffraction data were processed using autoPROC [30]. The phase problem of theWIPI3/ATG16L1 complex was solved by the molecular replacement method using the apo-form WIPI3 structure (PDB ID: 6IYY) [31]. The final refinement statistics of the solved structures in this study are listed in Table 1. All the structural diagrams were generated using PyMOL 2.6.0 software.

### 2.6. Co-Immunoprecipitation

Flag-tagged WIPI3 and mEGFP-tagged ATG16L1 plasmids (wild-type or mutants) were co-transfected into HEK293T cells using Lipofectamine 2000 transfection reagent (Thermo Fisher Scientific, USA). Cells were collected 36 h after transfection and lysed in the ice-cold cell lysis buffer containing a 50 mM Tris (pH = 7.5), 150 mM NaCl, 0.5% NP-40, 1 mM PMSF, 1% protease inhibitor cocktail for 40 min at 4 °C. Meanwhile, anti-GFP mAb-Agaroses (Medical & Biological Laboratories, Japan) were incubated by the ice-cold cell lysis buffer. Cell lysates were centrifuged at 14,500× *g* for 15 min at 4 °C to remove cell debris while anti-GFP mAb-Agaroses were centrifuged at 800× *g* for 3 min at 4 °C to remove the ice-cold cell lysis buffer. The supernatants of cell lysates were applied to the anti-GFP mAb-Agaroses for 1 h at 4 °C. The beads and non-bound ingredients were separated by centrifugation at 800× *g* for 3 min at 4 °C. After washing twice with the cold wash buffer containing 50 mM Tris (pH = 7.5), 150 mM NaCl, and 0.1% NP-40, the beads were re-suspended with the 1X SDS-PAGE sample buffer and boiled for 10 min at 65 °C. The prepared samples were analyzed by the SDS-PAGE assay and subsequently transferred onto the methanol-activated PVDF membrane (Millipore, USA). Western blot analysis was employed to detect the mEGFP-tagged ATG16L1 and Flag-tagged WIPI3 using a specific GFP antibody (Proteintech, China, catalog no. 66002-1-Ig, 1:2000 dilution) and Flag antibody (Proteintech, catalog no. 66008-4-Ig, 1:2000 dilution), respectively.

## 3. Results

### 3.1. WIPI3 Can Directly Interact with ATG16L1 but Not ATG5 and ATG12

To elucidate the molecular basis underlying the specific interaction between WIPI3 and the ATG16L1 complex, we constructed full-length ATG12 and ATG5 as well as the loop-truncated WIPI3 based on a previous truncation strategy utilized for structural characterizations of WIPI3 [21,22]. However, we retained the highly conserved WIPI3(75–80) region in our core WD40 construct of WIPI3 (hereafter referred to as WIPI3^Δ^), in order to avoid potential structural perturbations (Figure 1B and Figure S1). Using size exclusion chromatography (SEC)-based assays, we revealed that the central region of ATG16L1, the ATG16L1(78–247) fragment, can directly interact with WIPI3 but not ATG5 or ATG12 (Figure 1C and Figure S3).

### 3.2. Biochemical Mapping of the WIPI3-Interacting Region Within ATG16L1

Next, we sought to delimit the minimal WIPI3-binding boundary of ATG16L1 for further structural study. Due to the weak heat change observed between WIPI3 and ATG16L1(78–247) in our isothermal titration calorimetry (ITC) assays (Figure S4A,B), we performed the SEC-based assay as an alternative to qualitatively compare the interactions of WIPI3^Δ^ with four ATG16L1 fragments (residues 78–206, 78–197, 124–197, and 124–188), which have been characterized in our previous work [14]. Compared to the ATG16L1(78–247) fragment, both ATG16L1(78–197) and ATG16L1(78–206) fragments induced similar peak shifts of WIPI3^Δ^ (Figure S4C,D). Further removal of the N-terminal flexible ATG16L1(78–123) region does not have an obvious effect on the WIPI3^Δ^/ATG16L1 interaction, as both ATG16L1(124–197) and ATG16L1(124–188) fragments can interact well with WIPI3^Δ^ (Figure S4E,F). Taken together, we concluded that the WIPI3-interacting region (W3IR) of ATG16L1 is located within the coiled-coil domain of ATG16L1.

### 3.3. The Overall Structure of WIPI3^Δ^ in Complex with ATG16L1

Then, we aimed to structurally characterize the binding between WIPI3 and ATG16L1. Our initial attempts to crystallize the WIPI3^Δ^/ATG16L1(124–197) complex were unsuccessful. However, we managed to obtain good crystals that diffracted to 2.77 Å resolution using the WIPI3^Δ^/ATG16L1(124–188) complex. Through the molecular replacement method with the structure of the *apo*-form WIPI3 (PDB ID: 6IYY), we solved the crystal structure of the WIPI3^Δ^/ATG16L1(124–188) complex (Table 1). In the final complex structural model, each asymmetric unit contains two WIPI3 molecules and one ATG16L1(124–188) dimer, which assemble into a unique 2:2 stoichiometric hetero-tetramer (Figure 1D,E). As anticipated, two ATG16L1(124–188) molecules mainly form two continuous α-helices and wrap around each other, forming a parallel coiled-coil dimer (Figure 1D,E). Two WIPI3 molecules in the hetero-tetramer complex adopt highly similar seven-bladed β-propeller architectures (Figure S5A), akin to that of the *apo*-form yeast Hsv2 (Figure S5B), and symmetrically bind to the N-terminal opposite homo-dimeric interfaces of ATG16L1(124–188), each burying ~598 Å^2^ surface area (Figure 1D,E). Notably, blades 1 and 2 of WIPI3 in the WIPI3^Δ^/ATG16L1 complex exhibit a prominent twist compared to the previously reported structure of WIPI3 (Figure S5C), in which the conserved WIPI3(75–80) region is deleted [21]. Actually, the hydrophobic side chain of Y77 and the aliphatic side chain of K76 located in the WIPI3(75–80) region form extensive hydrophobic contacts with the hydrophobic side chains of F49, L68, W85, and M83 as well as the methyl group of T92 from WIPI3 (Figure S5D). Accordingly, the observed twist of the WIPI3 structure should be attributed to the removal of this highly conserved WIPI3(75–80) region.

### 3.4. The Molecular Interface in the WIPI3^Δ^/ATG16L1 Complex Structure

**Figure 2:**
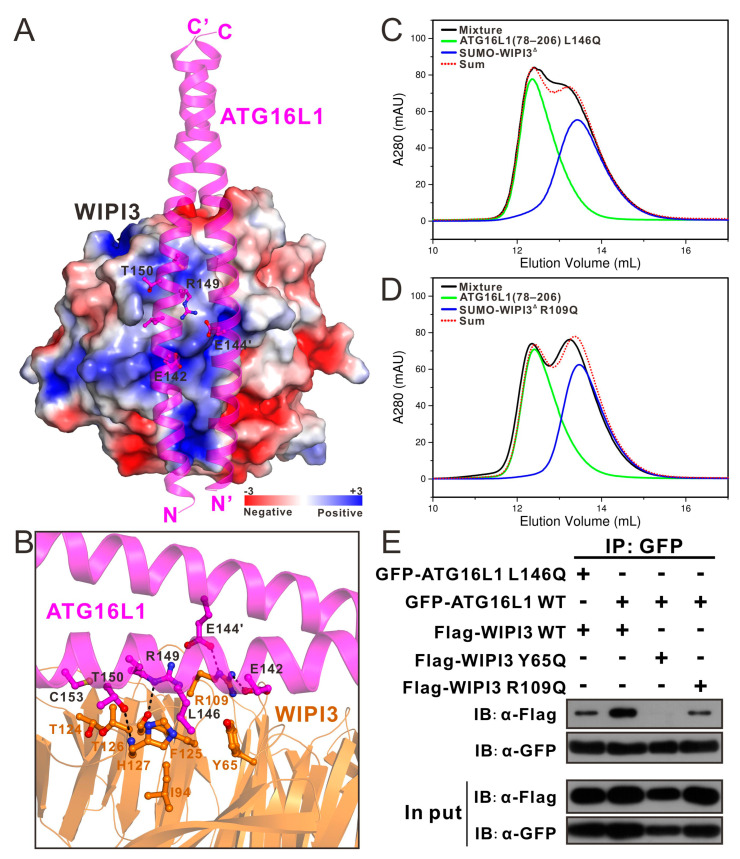
The molecular interface between the WIPI3 and ATG16L1 complex structure. (**A**) The combined surface charge representation and the ribbon-stick model showing the charge–charge interactions between WIPI3^Δ^ and ATG16L1. (**B**) The ribbon-stick model showing the detailed interactions between WIPI3^Δ^ and ATG16L1. The hydrogen bonds and salt bridges involved in the binding are shown as dotted lines. (**C**) Size exclusion chromatography-based analyses of the interactions of ATG16L1(78–206) L146Q mutant with SUMO-tagged WIPI3^Δ^. (**D**) Size exclusion chromatography-based analyses of the interactions of ATG16L1(78–206) with SUMO-tagged WIPI3^Δ^ R109Q mutant. (**E**) Co-immunoprecipitation assays showing that point mutations of key interface residues of WIPI3 or ATG16L1 observed in the WIPI3^Δ^/ATG16L1 complex structure decrease or essentially disrupt their specific interaction in cells. “IB” stands for immunoblotting.

Further comprehensive structural analysis uncovered that the molecular interface between WIPI3^Δ^ and the dimeric ATG16L1 is primarily constituted by residues from blades 2 and 3 of WIPI3, through a combination of polar and hydrophobic interactions (Figure 2A,B). In particular, the negatively charged E142 residue from one helical ATG16L1 chain and E144 from another chain of the ATG16L1 dimer couple with the positively charged R109 residue of WIPI3 to form charge–charge interactions and hydrogen bonds (Figure 2A,B). In addition, the side chains of R149 and T150 residues of ATG16L1 form hydrogen bonds with F125 and H127 residues of WIPI3, respectively (Figure 2B). Concurrently, the hydrophobic side chain of ATG16L1 L146 occupies the hydrophobic groove formed by the side chains of Y65, I94, F125, and H127 residues from WIPI3 (Figure 2B). Moreover, the methyl groups of T124 and T126 from WIPI3 form hydrophobic contacts with the hydrophobic side chain of C153 of ATG16L1 (Figure 2B). Notably, although all these key interface residues of WIPI3 are highly conserved during evolution (Figure S1), interface residues of ATG16L1 are merely conserved in primates and rodents (Figure S2). The specific interactions between WIPI3 and ATG16L1, as observed in the WIPI3^Δ^/ATG16L1 complex structure, were further validated using SEC-based assays. In accordance with the aforementioned structural data, individual point mutations of key residues involved in the binding interface of the WIPI3^Δ^/ATG16L1 complex, either from WIPI3 or ATG16L1, such as the Y65Q, disease-associated R109Q [32], and T126R mutations of WIPI3, or the E142R, E144R, and L146Q mutations of ATG16L1(78–206), all resulted in a significant reduction or a complete loss of the specific interaction between WIPI3^Δ^ and ATG16L1(78–206) (Figure 2C,D and Figure S6). Consistently, further co-immunoprecipitation assays demonstrated that point mutations of key interface residues of WIPI3 or ATG16L1, including the Y65Q and disease-associated R109Q mutations of WIPI3 and the L146Q mutation of ATG16L1, completely disrupted or largely decreased the specific association of the full-length WIPI3 and ATG16L1 in cells (Figure 2E). Notably, the critical ATG16L1-binding residues in WIPI3, such as Y65, T126, and H127 are not conserved in other WIPI family proteins, suggesting a unique binding mode adopted by WIPI3 for binding to ATG16L1 (Figure S7).

### 3.5. The Relationship of WIPI3 and WIPI2 in Binding to ATG16L1

**Figure 3:**
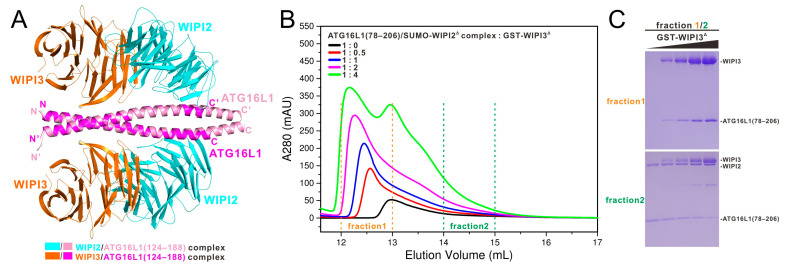
The relationship of WIPI3 and WIPI2 in associating with ATG16L1. (**A**) The ribbon diagram showing the structural comparison of the WIPI3/ATG16L1 and WIPI2/ATG16L1 complexes. In this drawing, the WIPI3 molecules in the WIPI3/ATG16L1 complex are shown in orange, while the WIPI2 molecules in the WIPI2/ATG16L1 complex are shown in cyan. ATG16L1 molecules are shown in magenta and pink, respectively. (**B**) Size exclusion chromatography analyses of the ATG16L1(78–206)/SUMO-WIPI2Δ complex incubated with an increasing molar ratio of GST-tagged WIPI3Δ proteins. Here, WIPI2Δ represents WIPI2b, including residues 13–362 but without residues 265–297. (**C**) The SDS-PAGE combined with Coomassie-blue staining analyses of the protein components of the indicated “fraction 1” and “fraction 2” fractions collected from the size exclusion chromatography experiments at different molar ratios of GST-WIPI3Δ in panel (**B**).

Further structural comparison analysis revealed that ATG16L1 packs extensively with the groove between blades 2 and 3 of WIPI3, similar to the binding mode of WIPI2 with ATG16L1 but with a different orientation for the bound ATG16L1 helix (Figure S5E,F). Although ATG16L1 utilizes completely different interface residues for binding to WIPI3 and WIPI2 (Figure S2), ATG16L1 actually adopts a similar strategy to interact with WIPI3 and WIPI2. Particularly, the key triad residues of ATG16L1 for binding to WIPI3 (E142, E144, and L146) and WIPI2 (D164, E165, and I171) are equally composed of two negatively charged residues and a hydrophobic residue [14]. Differently, the two negatively charged WIPI3-interacting residues (E142 and E144) are located in two distinct chains of ATG16L1 coiled-coil (Figure 2B). Importantly, structural comparison analysis showed that ATG16L1 coiled-coil is unable to simultaneously interact with WIPI3 and WIPI2 due to potential steric exclusions (Figure 3A). Indeed, our SEC-based assays coupled with SDS-PAGE analyses revealed that WIPI3 and WIPI2 are competitive in binding to ATG16L1(78–206) (Figure 3B,C).

### 3.6. The Relationship of ATG16L1 and ATG2 in Binding to WIPI3

**Figure 4:**
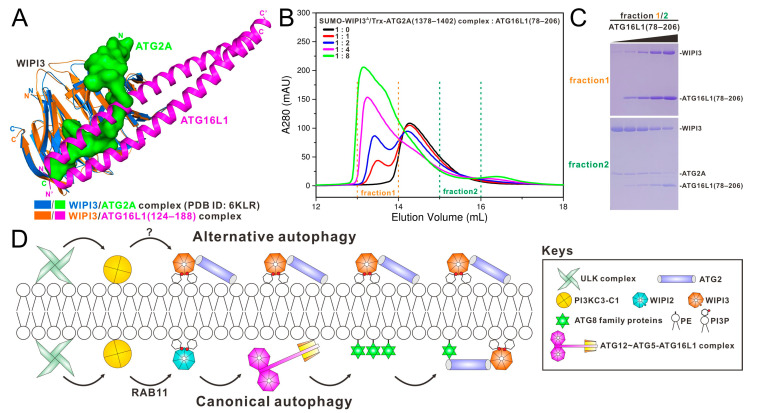
The relationship of ATG16L1 and ATG2 in binding to WIPI3. (**A**) The combined surface representation and the ribbon model showing the structural comparison of the WIPI3/ATG16L1 complex and the WIPI3/ATG2A complex (PDB ID: 6KLR). In this drawing, ATG16L1 molecules are shown in magenta, while ATG2A is shown in green with the surface representation. The WIPI3 molecules in the WIPI3/ATG16L1 and WIPI3/ATG2A complexes are shown in orange and marine, respectively. (**B**) Size exclusion chromatography analyses of the SUMO-WIPI3^Δ^/Trx-ATG2A(1378–1402) complex incubated with an increasing molar ratio of ATG16L1(78–206) proteins. (**C**) The SDS-PAGE combined with Coomassie-blue staining analyses of the protein components of the indicated “fraction 1” and “fraction 2” fractions collected from the size exclusion chromatography experiment at different molar ratios of ATG16L1(78–206) in panel (**B**). (**D**) A proposed cartoon model depicting the working mode of WIPI3 and ATG16L1 in alternative autophagy and canonical autophagy.

In addition to interacting with ATG16L1, WIPI3 can also directly bind to ATG2 [22]. To investigate whether ATG16L1 binding might influence the association between WIPI3 and ATG2, we performed relevant structural comparison analyses. The results demonstrated that despite the distinct binding modes adopted by ATG16L1 and ATG2 in their interaction with WIPI3, they nonetheless occupy the same WIPI3 pocket (Figure 4A). Notably, WIPI3 utilizes a multitude of identical interface residues to interact with both ATG16L1 and ATG2, such as Y65, I94, R109, F125, and H127 (Figure 2B and Figure S1). Therefore, ATG16L1 and ATG2 should be mutually exclusive in binding to the same WIPI3 molecule. Indeed, using SEC-based assays coupled with SDS-PAGE analyses, we confirmed that ATG16L1(78–206) and ATG2A(1378–1402) are unable to interact with WIPI3 simultaneously (Figure 4B,C).

## 4. Discussion

WIPI family proteins make distinct contributions to the canonical autophagy process. Particularly, WIPI2 bridges the PI3P signal from PI3KC3-C1 and the ATG8 PE-lipidation signal from the ATG16L1 complex, which is indispensable for phagophore biogenesis and elongation in canonical autophagy [11,13,14,33]. Meanwhile, WIPI1 depends on WIPI2 for proper localization to the phagophore, and further favors WIPI2-bridged autophagy flux [15]. WIPI3 and WIPI4 function downstream and redundantly in complex with ATG2 for supplying lipid sources, and also regulate the autophagosome–lysosome fusion together with EPG5, especially in neural cells [15,34]. In this work, we discovered that WIPI3 can directly interact with the coiled-coil domain of ATG16L1 (Figure 1). Importantly, we determined the high-resolution structure of WIPI3 in complex with the ATG16L1 coiled-coil domain, and unraveled the molecular basis underlying the specific interaction of WIPI3 with ATG16L1 as well as a previously unknown binding mode between ATG16L1 and WIPI family proteins. Interestingly, the WIPI3/ATG16L1 interaction is likely to be dispensable in canonical autophagy, since even the deprivation of WIPI3 has no obvious impact on the canonical autophagy process [28,33]. However, we cannot rule out the potential functional roles of WIPI3-mediated recruitment of the ATG16L1 complex in other types of autophagy pathways. Notably, our biochemical and structural results demonstrated that WIPI3 and WIPI2 are competitive in binding to ATG16L1 coiled-coil (Figure 3). In contrast to WIPI2, yeast ATG21 enabled the recruitment but not the activation of human ATG16L1 complex [11]. Actually, yeast ATG21 was recently reported to interact with ATG16L1 WBS1 but not WBS2 that is located in ATG16L1 coiled-coil (Figure 1A) [35], implying that the specific interaction between WIPI2 and ATG16L1 coiled-coil mediates the activation of the ATG16L1 complex. Based on our studies together with other previous reports [11], we inferred that WIPI3 can directly recruit and activate the ATG16L1 complex by binding to the coiled-coil domain of ATG16L1. Therefore, it is highly possible that the membrane proximity of ATG16L1 coiled-coil might pull the N-terminal ATG5~ATG12 conjugates close to the membrane surface, thereby facilitating the lipidation of ATG8 family proteins (Figure S8). Notably, during CASM, the recruitment of the ATG16L1 complex is not only dependent on the ATG16L1 WD40 domain but also the coiled-coil region of ATG16L1 [20]. In the future, it will be interesting to know whether CASM entails a specific membrane-binding adaptor to interact with ATG16L1 coiled-coil for the activation of the ATG16L1 complex.

WIPI3 plays an indispensable role in maintaining neural autophagy and cognitive function, whose depletion results in cerebellar atrophy and neurodegeneration [28,36]. Notably, the R109Q missense mutation of WIPI3 is closely related to intellectual disability [32]. In this study, based on biochemical and structural analyses (Figure 2B,D), we showed that the disease-associated R109Q mutation of WIPI3 significantly reduces the interaction between WIPI3 and ATG16L1. Interestingly, the disease-associated WIPI3 R109Q mutation was also reported to impair the binding between WIPI3 and ATG2 [22]. Given that the R109Q mutation of WIPI3 simultaneously impairs the interactions of WIPI3 with ATG2 and ATG16L1, further research is required to elucidate the precise etiology of WIPI3 R109Q-induced diseases.

Our structural modeling and biochemical analyses revealed that WIPI3 is unable to simultaneously interact with ATG16L1 and ATG2 (Figure 4A–C). Although in vitro reconstitution studies clearly showed that WIPI3 can replace WIPI2 to fulfill the positive feedback with PI3KC3-C1 and promote the lipidation of ATG8 family proteins [11], it is the knockout of WIPI2 instead of WIPI3 that inhibits the cellular lipidation in canonical autophagy [13,28,33]. Meanwhile, undelivered Golgi cargoes are degraded upon PI3KC3-C1-dependent WIPI3, but not WIPI2 or the ATG16L1 complex translocation to the Golgi apparatus in alternative autophagy [28]. Hence, PI3KC3-C1 is necessary but insufficient for the recruitment of either WIPI2 or WIPI3 in vivo. Actually, PI3KC3-C1 and RAB11 work in concert on WIPI2-mediated canonical autophagy [37]. Based on the aforementioned facts together with our results in this study, we proposed a model to illustrate the working mode of canonical autophagy and alternative autophagy (Figure 4D). During the canonical autophagy, RAB11 permits the positive feedback between PI3KC3-C1 and WIPI2 (Figure 4D). Meanwhile, the ATG16L1 complex is saturated by excessive WIPI2 and deprived of the opportunity to interact with WIPI3. As a result, phagophore elongation in canonical autophagy relies on the associations between lipidated ATG8 family proteins and the WIPI3/4-ATG2 complex (Figure 4D). In contrast, an unknown factor might enable the positive feedback between PI3KC3-C1 and WIPI3 in alternative autophagy (Figure 4D). Due to the tight associations of WIPI3 with ATG2 as well as ATG16L1 with WIPI2, WIPI3 has no chance to access the ATG16L1 complex in alternative autophagy. Therefore, there is no subsequent lipidation of ATG8 family proteins. Consequently, phagophore expansion in alternative autophagy is supported by the direct translocation of the WIPI3-ATG2 complex (Figure 4D), thereby bypassing the prerequisite of the decorated ATG8 family proteins.

## Figures and Tables

**Table 1:** Statistics for data collection and processing, structure solution, and refinement of the crystal structure of the WIPI3/ATG16L1 complex.

Crystal		The WIPI3/ATG16L1 Complex
PDB accession code		8ZQG
**Data collection and processing**		
Diffraction source		Synchrotron
Detector		DECTRIS EIGER2 S 9M
Wavelength (Å)		0.97918
Spacegroup		*P*6_3_
Cell dimensions	a, b, c (Å)	182.66, 182.66, 54.34
	α, β, γ (^o^)	90.00, 90.00, 120.00
Resolution range (Å)		91.33–2.77 (2.82–2.77)
No. of total reflections		459,708 (22,802)
Multiplicity		17.2 (17.1)
No. of unique reflections		26,763 (1330)
Completeness (%)		100.0 (100.0)
Mean I/σ(I)		16.1 (2.1)
Wilson B factor (Å^2^)		76.23
R-merge		0.143 (1.755)
R-meas		0.147 (1.808)
CC1/2 (%)		99.6 (62.2)
**Structure solution and refinement**		
R-work (%)		18.99 (27.95)
R-free (%)		23.26 (33.89)
R.m.s. deviations	bonds (Å)	0.008
	angles (^o^)	1.216
Average B-factor (Å^2^)		85.60
Rotamer outliers (%)		0.49
Clashscore		8.28
Ramachandran plot (%)	most favored	98.33
	additionally allowed	1.67
	outliers	0.00

Numbers in parentheses represent the value for the highest-resolution shell.

## Data Availability

The coordinates and structure factors of the WIPI3^Δ^/ATG16L1(124–188) complex solved in this study have been deposited in the Protein Data Bank under the accession code 8ZQG.

## Supplementary Materials

The following supporting information can be downloaded at https://www.mdpi.com/article/10.3390/cells13242113/s1, Figure S1: Structure-based sequence alignment analyses of WIPI3 from different species; Figure S2: Structure-based sequence alignment analyses of ATG16L1(78–247) from different species; Figure S3: Biochemical characterizations of the interactions of WIPI3^Δ^ with ATG5 and ATG12; Figure S4: Biochemical mapping of the boundary of ATG16L1 W3IR; Figure S5: Structural analyses of the WIPI3^Δ^/ATG16L1 complex; Figure S6: The validations of the key interface residues in the WIPI3^Δ^/ATG16L1 complex structure by size exclusion chromatography-based analyses; Figure S7: Sequence alignment analyses of four WIPI family proteins from human species; Figure S8: A proposed cartoon model depicting the working mode of ATG16L1 in the PE-lipidation process of ATG8 family proteins.
