# Supplementary material for: Structure of the WIPI3/ATG16L1 Complex Reveals the Molecular Basis for the Recruitment of the ATG12~ATG5-ATG16L1 Complex by WIPI3
Source: Cells. 2024 Dec 20;13(24):2113. doi: 10.3390/cells13242113 (PMC11727070; doi:10.3390/cells13242113)
Supplement: Supplementary file 1 [file cells-13-02113-s001.zip › cells-3306910-supplementary.pdf]

Supplementary Figures for:

Structure of the WIPI3/ATG16L1 complex reveals the molecular basis for the recruitment of the ATG12~ATG5-ATG16L1 complex by WIPI3

Xinyu Gong, Yingli Wang, Yuqian Zhou and Lifeng Pan*

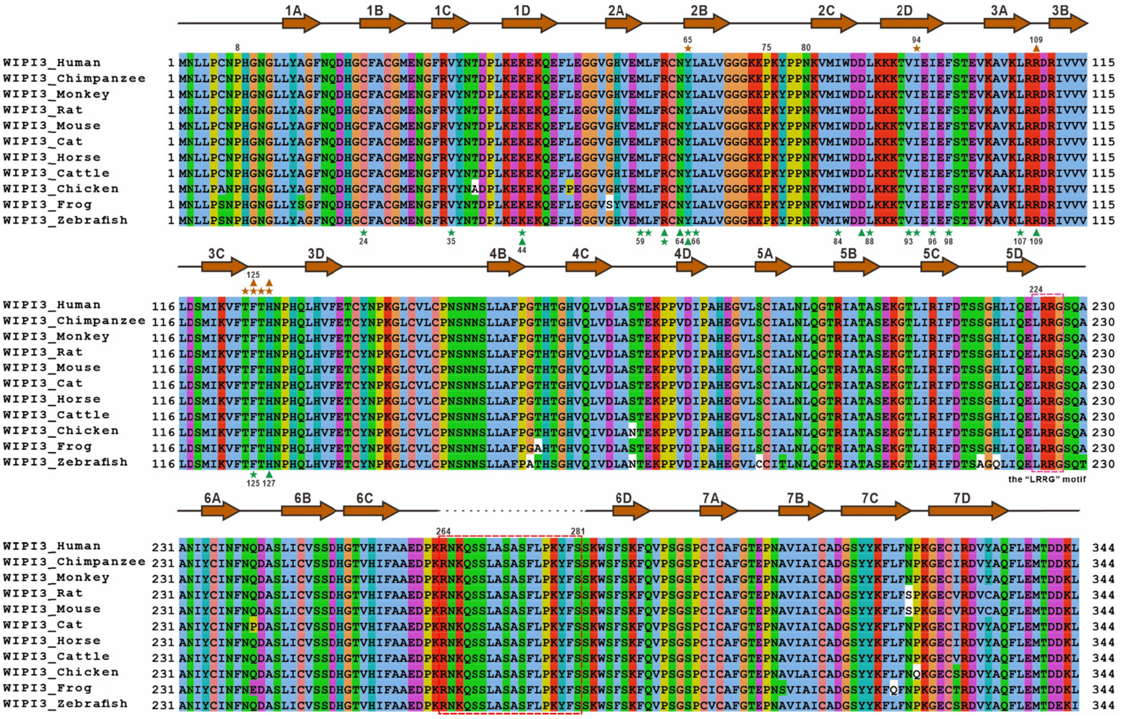

Figure S1. Structure-based sequence alignment analyses of WIPI3 from different species. In this alignment, the conserved residues are highlighted by colors using software Jalview 2.10.5 (http://www.jalview.org/). In addition, the conserved interface residues involved in the interactions with ATG16L1 and ATG2A are respectively highlighted with orange and green stars (hydrophobic interactions) or triangles (polar interactions). Meanwhile, the PI3P-binding “LRRG” motif is further boxed and highlighted.

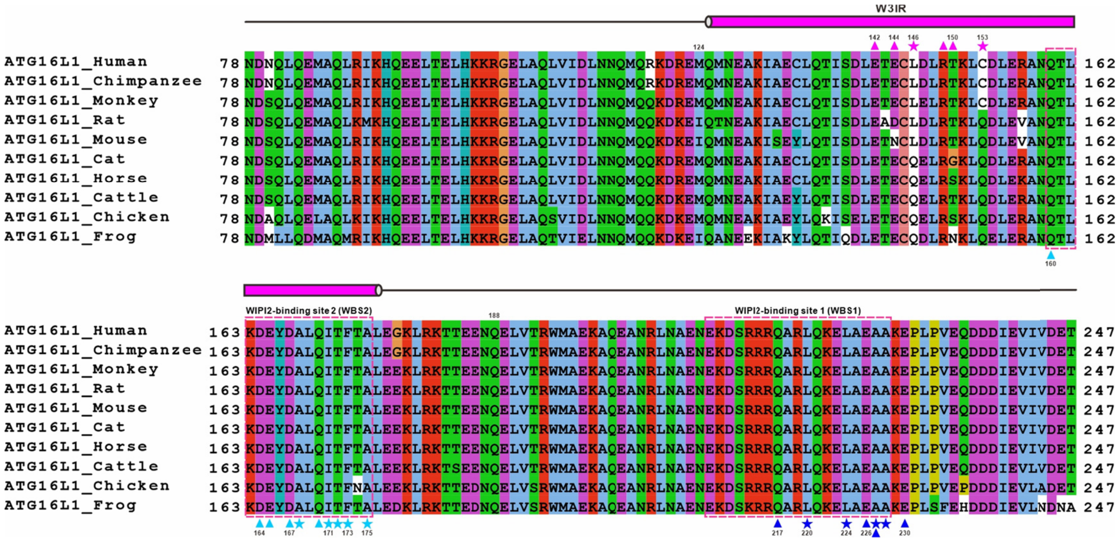

Figure S2. Structure-based sequence alignment analyses of ATG16L1(78-247) from different species. In this alignment, the conserved residues are highlighted by colors using software Jalview 2.10.5 (http://www.jalview.org/). Furthermore, the conserved interface residues of ATG16L1 involved in the interactions with WIPI3 are highlighted with magenta stars (hydrophobic interactions) or triangles (polar interactions). Meanwhile, the conserved interface residues of ATG16L1 WBS1 and WBS2 involved in the interactions with WIPI2 are respectively highlighted with sky bule and deep blue stars (hydrophobic interactions) or triangles (polar interactions).

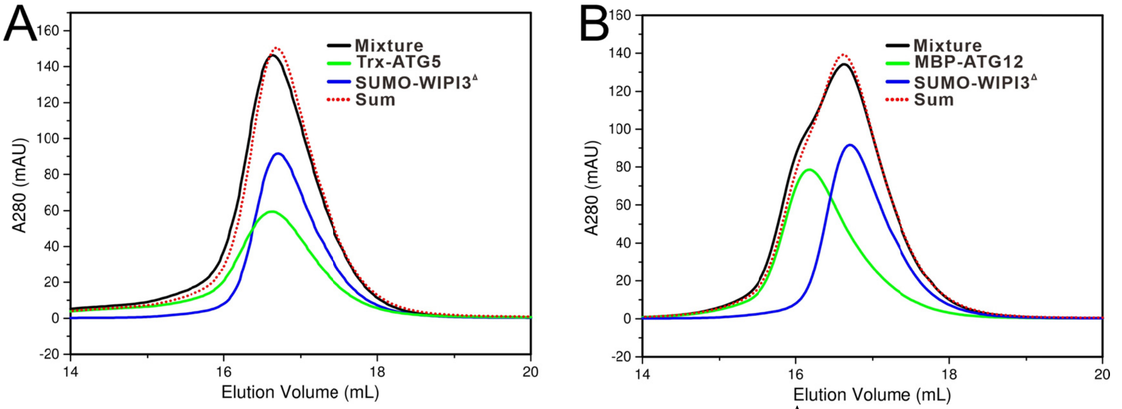

Figure S3. Biochemical characterizations of the interactions of WIPI3$^{\Delta}$ with ATG5 and ATG12. (**A** and **B**) Size exclusion chromatography analysis of the interactions of SUMO-tagged WIPI3$^{\Delta}$ with Trx-tagged ATG5 (**A**) and MBP-tagged ATG12 (**B**).

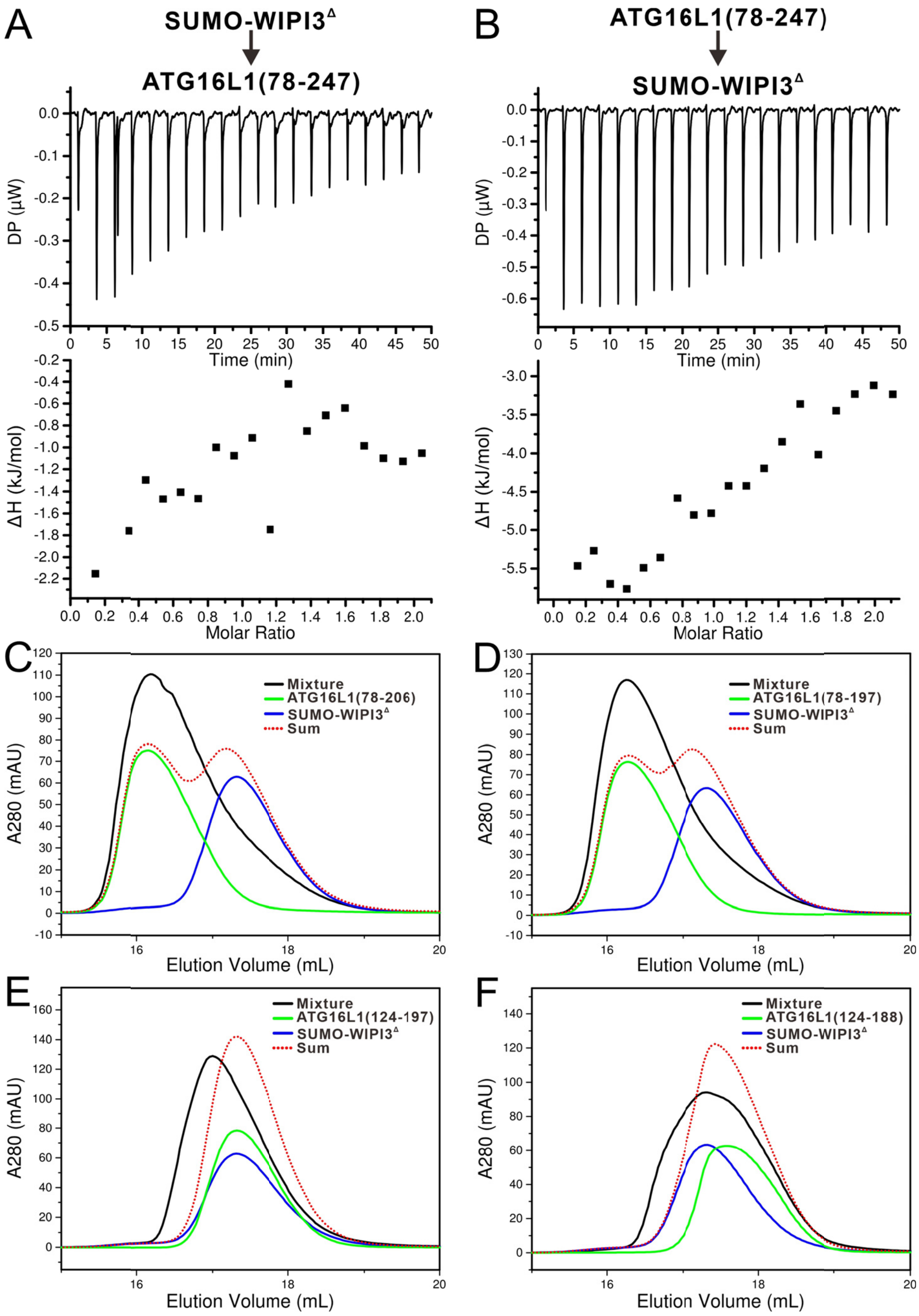

Figure S4. Biochemical mapping of the boundary of ATG16L1 W3IR. (**A** to **B**) ITC-based measurements of the binding affinities between SUMO-tagged WIPI3$^{\Delta}$ and ATG16L1(78-247). DP is the differential power measured by the ITC machine and ΔH is the enthalpy change measured by the ITC machine. (**C** to **F**) Size exclusion chromatography analysis of the interactions of SUMO-tagged WIPI3$^{\Delta}$ with ATG16L1(78-206) (**C**), ATG16L1(78-197) (**D**), ATG16L1(124-197) (**E**), and ATG16L1(124-188) (**F**).

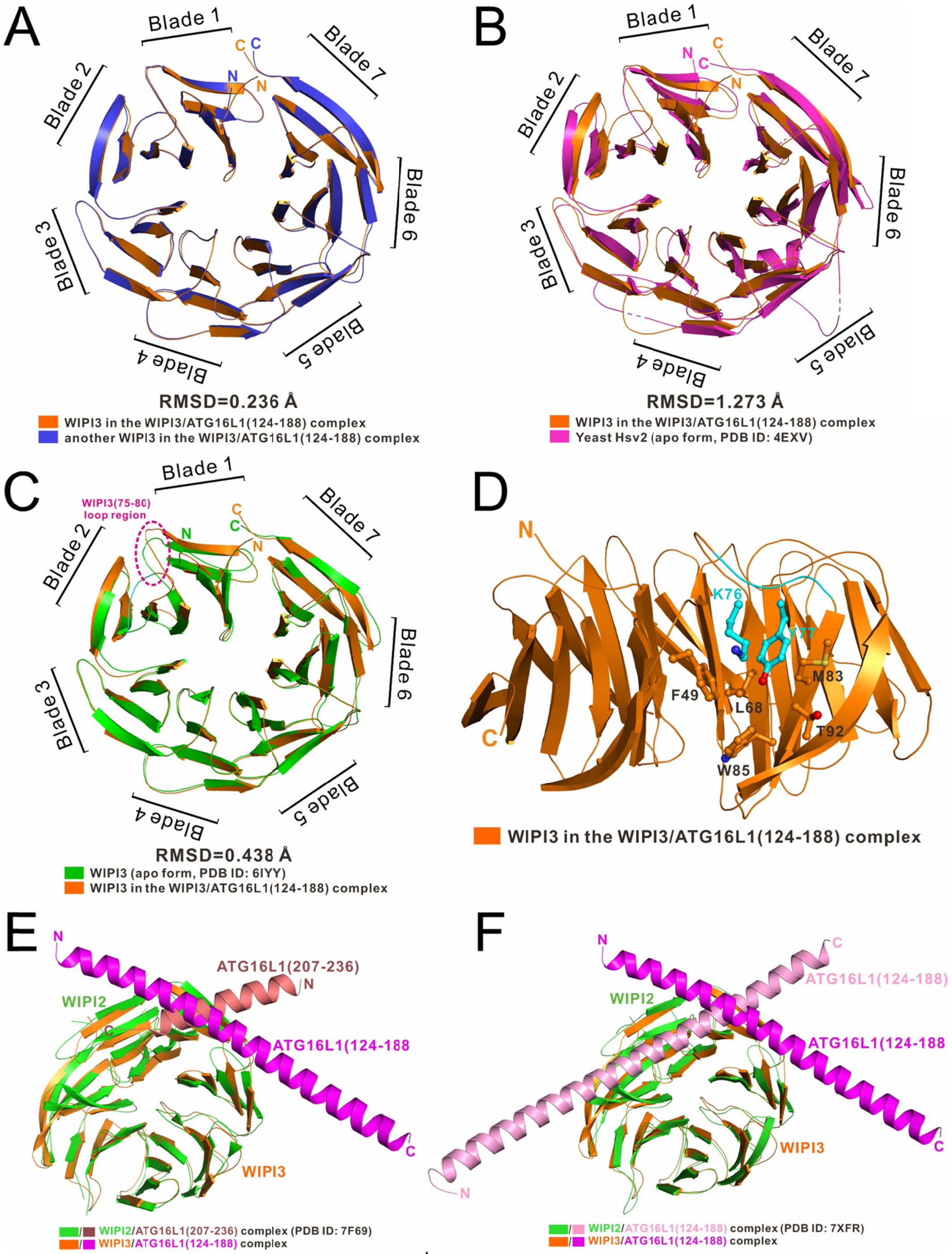

Figure S5. Structural analyses of the WIPI3$^{\Delta}$/ATG16L1 complex. (**A**) The ribbon diagram showing the structure comparison of two WIPI3 molecules in the WIPI3$^{\Delta}$/ATG16L1 complex that is colored in orange and blue, respectively. (**B**) The ribbon diagram showing the structure comparison of WIPI3 in the WIPI3$^{\Delta}$/ATG16L1 complex with the yeast Hsv2 (PDB ID: 4EXV). (**C**) The ribbon diagram showing the structure comparison of WIPI3 in the WIPI3$^{\Delta}$/ATG16L1 complex with the *apo*-form

WIPI3 (PDB ID: 6IYY). (**D**) The ribbon-stick model showing the detailed hydrophobic interactions between K76 and Y77 of WIPI3, and F49, L68, M83, W85 and T92 of WIPI3. (**E**) The ribbon diagram showing the structure comparison of the WIPI3$^{\Delta}$/ATG16L1(124-188) complex with the WIPI2$^{\Delta}$/ATG16L1(207-236) complex (PDB ID: 7F69). (**F**) The ribbon diagram showing the structure comparison of the WIPI3$^{\Delta}$/ATG16L1(124-188) complex with the WIPI2$^{\Delta}$/ATG16L1(124-188) complex (PDB ID: 7XFR).

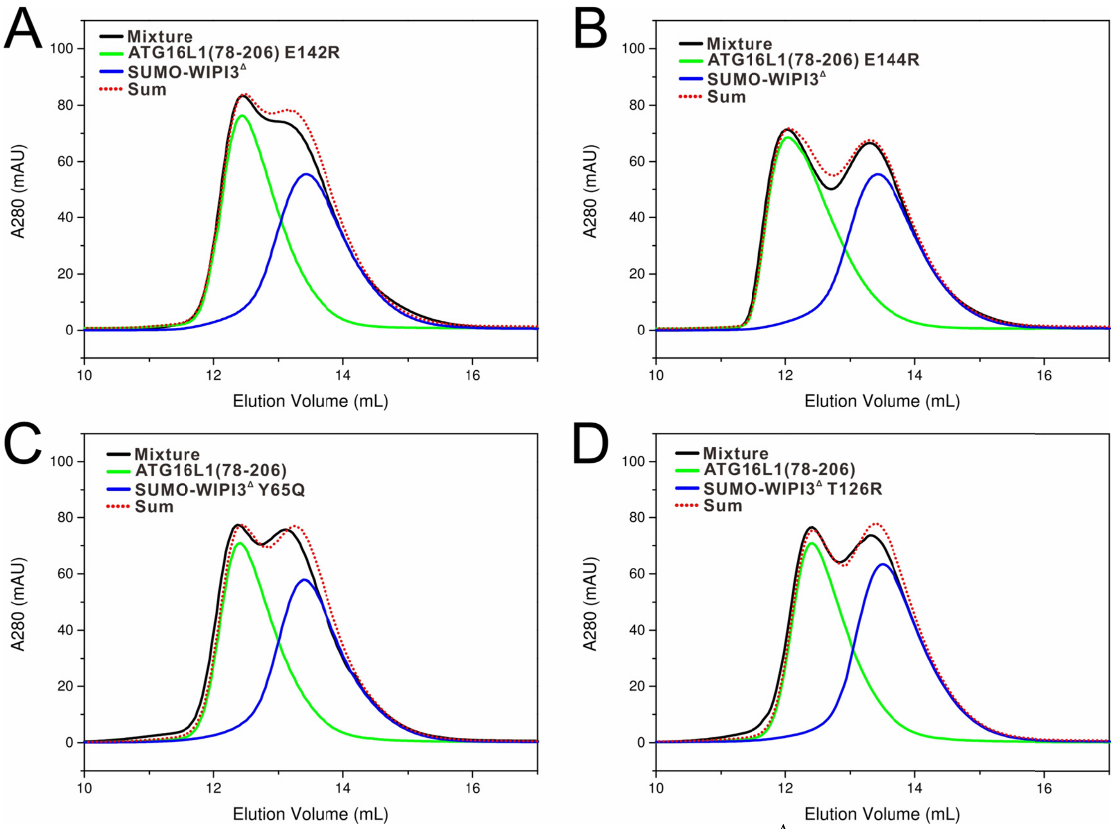

Figure S6. The validations of the key interface residues in the WIPI3$^{\Delta}$/ATG16L1 complex structure by size exclusion chromatography-based analyses. (**A** and **B**) Size exclusion chromatography-based analyses of the interaction of SUMO-tagged WIPI3$^{\Delta}$ with the ATG16L1(78-206) E142R mutant (**A**), or the ATG16L1(78-206) E144R mutant (**B**). (**C** and **D**) Size exclusion chromatography-based analyses of the interactions of ATG16L1(78-206) with the SUMO-tagged WIPI3$^{\Delta}$ Y65Q mutant (**C**), or the SUMO-tagged WIPI3$^{\Delta}$ T126R mutant (**D**).

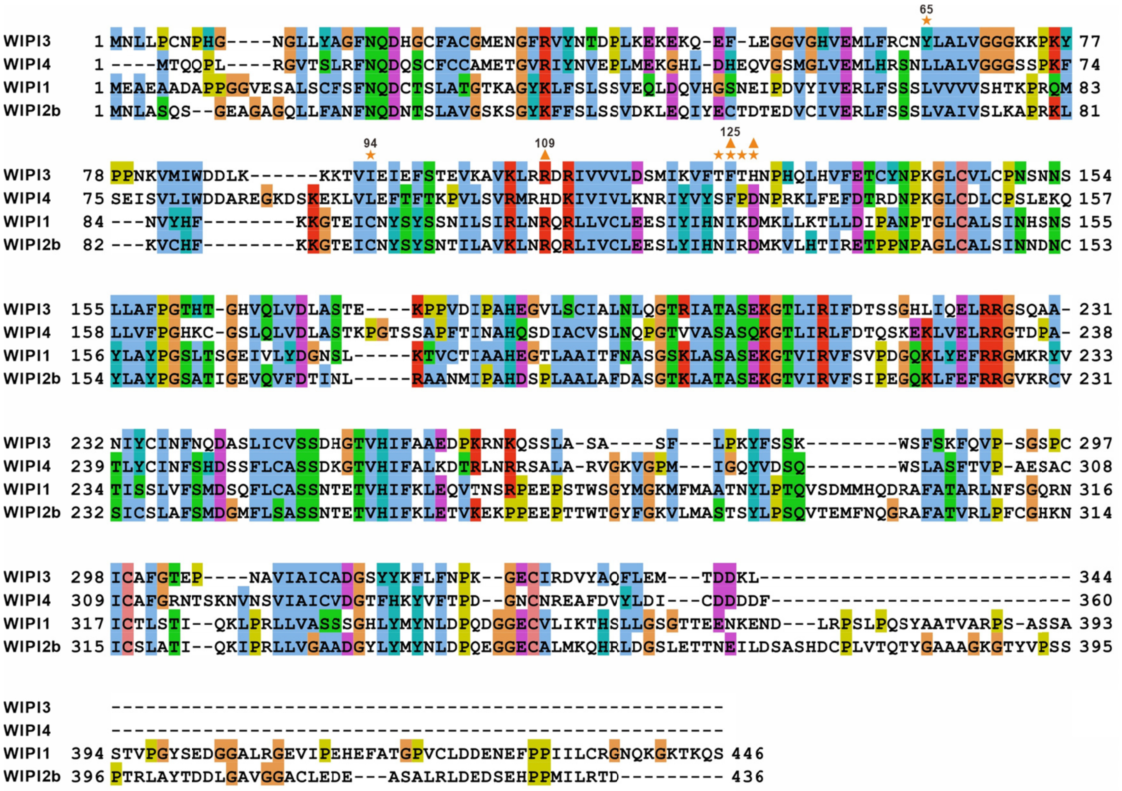

Figure S7. Sequence alignment analyses of four WIPI family proteins from human species. In this alignment, the conserved residues are highlighted by colors using software Jalview 2.10.5 (http://www.jalview.org/). Meanwhile, the interface residues involved in the interactions of WIPI3 with ATG16L1 are respectively highlighted with orange stars (hydrophobic interactions) or triangles (polar interactions).

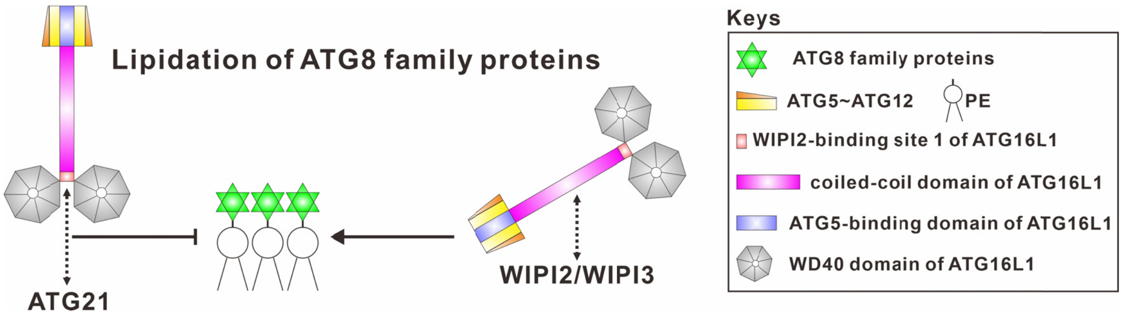

Figure S8. A proposed cartoon model depicting the working mode of ATG16L1 in the PE-lipidation process of ATG8 family proteins.
